# Learning from the Implementation of Disability-Inclusive Maternity Care: A Scoping Review

**DOI:** 10.3390/healthcare13182315

**Published:** 2025-09-16

**Authors:** Sarah Clifford, Meighan Mary, Briana Kramer, Mairead C. Minihane, Brina Ratangee, Erin M. Gilmer, Andreea A. Creanga

**Affiliations:** 1 Department of International Health, Johns Hopkins Bloomberg School of Public Health, Baltimore, MD 21205, USA; 2 International Center for Maternal and Newborn Health, Johns Hopkins Bloomberg School of Public Health, Baltimore, MD 21205, USA; 3 Department of Medicine, Health and Society, Vanderbilt University, Nashville, TN 37235, USA; 4 Independent Researcher, New Haven, CT 06510, USA; 5 Department of Obstetrics and Gynecology, Johns Hopkins School of Medicine, Baltimore, MD 21205, USA

**Keywords:** maternal health, disabilities, impairments, quality, implementation, pregnancy, postpartum

## Abstract

**Background/Objectives**: A growing body of evidence has revealed the multifaceted barriers populations with disabilities face during pregnancy and postpartum. This scoping review aimed to synthesize the literature on the implementation of disability-inclusive maternity care services for patients with disabilities. **Methods**: PubMed/MEDLINE, Embase, Scopus, PsycInfo were sourced for literature between January 2013 and July 2025. Articles were eligible for inclusion in the review if they reported on the implementation of interventions that aimed to improve quality of maternity care for pregnant or postpartum patients with physical, sensory, intellectual, mental impairments. In total, 6279 studies were screened to yield a final sample of 13 eligible articles. Key characteristics and implementation outcomes were extracted and synthesized from each eligible article. **Results**: Three of the studies targeted populations with physical impairments, five targeted populations with intellectual impairments, and five focused on populations with mental impairments. A variety of interventions were employed to improve the quality of care, targeting functional referral systems (*n* = 4), competent and motivated human resources (*n* = 4), actionable information systems (*n* = 3), effective communication (*n* = 1), and evidence-based practices (*n* = 1). Eligible studies reported acceptability (*n* = 11), adoption (*n* = 7), fidelity (*n* = 2), and penetration (*n* = 1) outcomes. The interventions generally reported high acceptability among providers and patients and emphasized the importance of participatory development and phased introduction. **Conclusions**: Significant gaps in the evidence remain across all categories of impairments. Additional research is needed to understand what types of interventions can be effectively implemented to improve the quality of maternity care for pregnant and postpartum patients with disabilities.

## 1. Introduction

Globally, one in five women experience disability and physical, sensory, intellectual, or mental impairments [1]. While people with disabilities (PwD) report similar pregnancy rates as those without disabilities [2,3], they are at a significantly higher risk of adverse maternal outcomes [4,5,6,7,8]. Compared to those without disabilities, pregnant PwD have eleven times the risk for maternal death, six times the risk of thromboembolism, four times the risk of cardiovascular events, three times the risk of infection, and twice the risk of severe preeclampsia [6]. Among U.S. hospital deliveries during 2016–2021, they were 1.9 times more likely to experience severe maternal morbidity or “near miss” complications than women without a disability [4].

International treaties enshrine the rights of PwD to the enjoyment of the highest attainable standard of health without discrimination; and mandate they are provided with the same range, quality, and standard of health care services and programs as their non-disabled counterparts [9]. However, patients living with disabilities report varying levels of satisfaction with perinatal services and face challenges in receiving high quality, respectful, and inclusive maternity care [10]. Research has identified multi-level barriers, including: systems barriers and fragmented care delivery [11,12,13,14], informational barriers and a lack of effective communication with health care professionals [15,16,17,18,19,20,21], physical barriers and inaccessible health infrastructure or equipment [13,22,23,24,25], and attitudinal barriers related to health workers’ limited knowledge or biases towards caring for PwD [14,16,26,27,28,29,30,31].

In light of growing evidence, researchers and professional organizations are increasingly prioritizing maternal health outcomes and experiences for PwD. In May 2025, for instance, the American College of Obstetricians and Gynecologists published guidance calling on providers to improve access to obstetric care for PwD and facilitate services in a “safe and welcoming manner” [32]. More broadly, in November 2024, the World Health Organization (WHO) released their first disability inclusion guide to provide national level stakeholders with strategic recommendations to strengthen health service delivery for people with disabilities [33]. Both guidance documents are encouraging, but neither provide information on concrete provider or institution level interventions or implementation guidance to improve the quality of maternity care for PwD.

A 2014 systematic review exploring strategies to improve outcomes for PwD throughout the perinatal period found limited published research [34]. A decade later, we aim to expand on this review to understand advancements in developing, implementing, and testing innovative approaches to improve the quality of maternity care for PwD. Thus, this scoping review synthesizes the literature on implementation of disability-inclusive maternity care approaches for patients with physical, sensory, intellectual, and mental impairments.

## 2. Materials and Methods

A scoping review of peer-reviewed literature was conducted to understand the gamut of maternal health interventions and approaches employed to improve the quality of care for pregnant and postpartum patients with disabilities. PubMed/Medline, Embase, Scopus and PsycInfo were searched electronically in July 2024, and repeated in July 2025. An adapted search strategy was used, building on WHO’s International Classification of Functioning, Disability, and Health (ICF) definition of disability [35] and a previous literature search conducted by Malouf et al. (2014) [34]. To ensure all relevant literature was captured, the search strategy (Supplementary Materials S1) employed both general disability search terms and condition-specific search terms [36,37,38]. A protocol for this review was registered online with Open Science Framework [39]. The Preferred Reporting Items for Systematic Reviews and Meta-Analyses extension for Scoping Reviews (PRISMA-ScR) [40] was used for reporting study findings and the completed checklist is available in the Supplemental Materials (Supplementary Materials S2).

Table 1 outlines the eligibility criteria for inclusion in this review. The literature was eligible if it assessed implementation of a maternal health intervention for pregnant and postpartum patients with a pre-existing disability (physical, sensory, intellectual, or mental impairments) and reported on select implementation outcomes (acceptability, adoption, fidelity, penetration, and sustainability). Peer-reviewed literature from January 2013 (i.e., to include articles published after the literature search conducted by Malouf et al. for their 2014 review [34]) to July 2025 in English, French, or Spanish were eligible. Protocols, case reports, dissertations, and conference abstracts or posters were excluded.

Search results were uploaded to Covidence [42] for review. After duplicates were removed, title/abstract screening and full text review were conducted by two members of the team with a third team member resolving any discrepancies in screening decisions. Relevant reviews identified during screening were hand-searched for eligible studies.

Data were extracted from relevant articles using a piloted data collection template. Information was collected on the reference characteristics, study methodology, intervention details (name, description, implementation setting, population), and implementation outcomes. Studies were grouped by the population they targeted using categories of impairment from the UN Convention on the Rights of Persons with Disabilities (CRPD) (physical, sensory, intellectual, and mental) [9], as defined in Table 2.

Reviewers classified the primary aim of each intervention using the eight domains of WHO’s framework for the quality of maternal and newborn health care [41]: provision of care domains (evidence-based practices, actionable information systems, and functional referral systems), experience of care domains (effective communication, respect and preservation of dignity, and emotional support) as well as cross-cutting domains (competent and motivated human resources, and essential physical resources). Data on implementation outcomes were collected as defined in Table 3. Following extraction, a matrix was created to synthesize findings and display articles by population, type of intervention, and outcomes reported. Considering the aim of the scoping review was to provide an update on evidence, regardless of methodological quality, there was no critical appraisal or assessment of risk of bias among eligible articles [40].

## 3. Results

**Figure 1:**
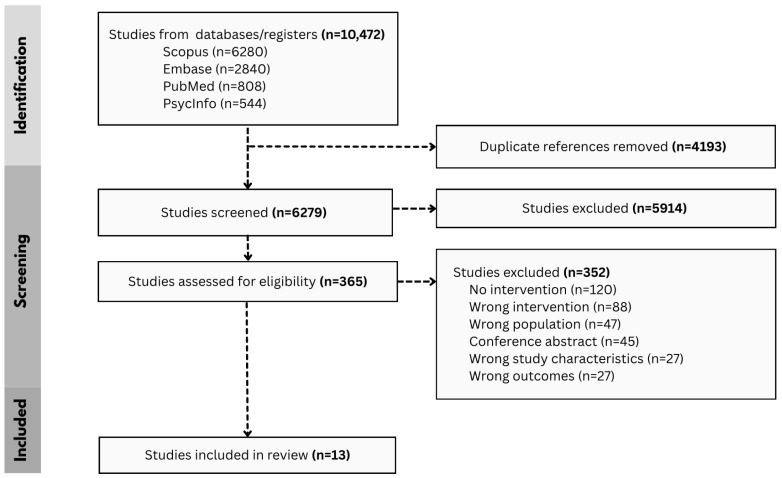
PRISMA Flow Diagram.

The search results identified 10,472 articles (Figure 1). After uploading to screening software, 4193 duplicates were removed, 6279 were screened, and 365 underwent full text review. During full-text review, 352 studies were excluded based on the following criteria: 120 did not report on an intervention, 88 reported on an intervention that did not aim to improve a domain of quality of maternity care, 45 were conference abstracts, 47 were not focused on populations with disabilities, 27 were the wrong study or article type, and 25 did not report on outcomes of interest. No additional studies were found through hand-searching of systematic literature reviews identified in screening.

Following review, 13 studies were found to be eligible (Table 4) [45,46,47,48,49,50,51,52,53,54,55,56,57]. Of these articles, 23% (3/13) focused on populations with physical impairments [45,46,47], 38% (5/13) focused on populations with intellectual impairments [48,49,50,51,52], and 38% (5/13) focused on populations with mental impairments [53,54,55,56,57]. More than half of the studies were conducted in Europe [46,47,48,49,50,51,52], three were conducted in North America [45,53,54], two in the Asia-Pacific region [55,56], and one in the Middle East and North African region [57]. A majority of the studies were published since 2020 [45,46,47,50,51,52,53,57].

**Figure 2:**
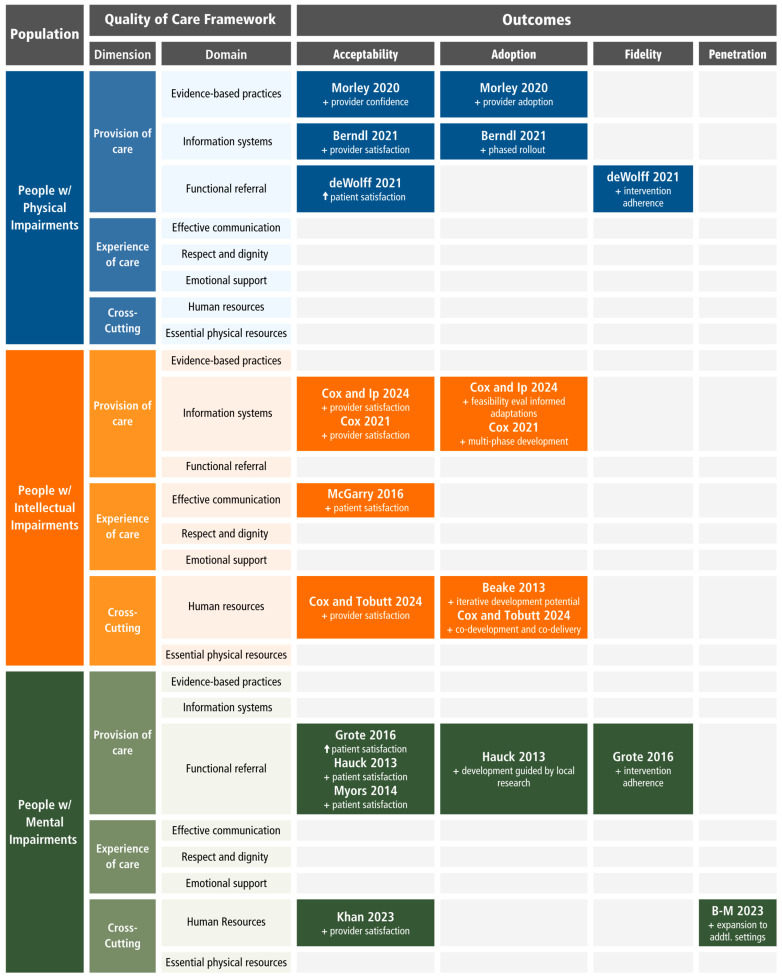
Outcomes Reported by Population and Primary Intervention Domain. Key: + = Beneficial; ↑ = Increase [45,46,47,48,49,50,51,52,53,54,55,56,57].

The studies employed diverse methodologies, including qualitative methods [47,49,50,55,56], mixed-methods [48,51,52,53], randomized controlled trials [46,54], and cross-sectional design [45]. One article was a commentary [57]. Most of the studies had small sample sizes and were pilots (Table 4). Acceptability [45,46,47,49,50,51,52,53,54,55,56], and adoption [45,47,48,50,51,52,56] outcomes were reported across all populations with physical, intellectual, and mental impairments. Fidelity was reported for interventions in two populations (physical and mental impairments) [46,54], and penetration was reported in one intervention for women with mental health conditions [57]. No interventions reported on sustainability outcomes (Figure 2).

The 13 eligible articles reported on 12 unique interventions. Four attempted to improve the quality of care by primarily addressing functional referral systems [46,54,55,56], four primarily addressed competent and motivated human resources [48,52,53,57], two actionable information systems [45,50,51], one effective communication [49], and one evidence-based practices [47]. More than half of the interventions (66%) were implemented during pregnancy (8/12) [45,47,48,50,51,53,54,56,57] and the remaining 33% were implemented through the postpartum period (4/12) [46,49,52,55]. The location and mode of intervention delivery varied across interventions. Most were implemented fully in-person, but two interventions could be delivered by phone [54,55]. Around one-third (33%, 4/12) were implemented in multiple settings, including health facilities and community environments [47,50,51,54,55]. Interventions taking place in a single facility were mostly implemented in tertiary care hospitals or specialty units embedded within them [45,46,48,56,57]. Two interventions were delivered in academic settings (medical school [53] and midwifery school [52]), and one intervention did not report the setting of delivery [49].

### 3.1. Physical Impairments

A quarter of the interventions identified in our search targeted populations with physical impairments (i.e., functional difficulties and mobility challenges) [45,46,47] (Table 4). One intervention focused on chronic medical conditions [46], one on epilepsy [47], and one article targeted people with physical disability broadly [45]. The interventions were delivered by midwives [46,47] and sonographers [45].

The studies employed interventions which aimed to improve actionable information systems, functional referral systems, evidence-based practices, and effective communication (Figure 1). Berndl and Khatib (2021) addressed actionable information systems at a specialized clinic for people with physical disabilities (PwPD), by having sonographers document their scans with logbooks which later informed quality improvement modifications to the unit [45]. DeWolff and team’s (2021) intervention, ChroPreg, used midwives to provide care coordination services to patients with chronic medical conditions [46]. Morley et al. (2020) developed a toolkit for midwives with guidance on evidence-based risk assessment and medication management for patients with epilepsy [47].

All studies focused on populations with physical impairments assessed acceptability; one focused on acceptability among patients [46], and two among providers [45,47]. DeWolff et al. (2021) measured satisfaction with maternity care using the Pregnancy and Childbirth Questionnaire and found recipients of the ChroPreg intervention were significantly more satisfied with their overall maternity care, personal treatment, and education compared than those receiving usual care (MD = 6.3; 95% CI: 3.0–10.0, *p* < 0.0001) [46]. Focusing on providers, Berndl and Khatib (2021) reported that sonographers found their QI intervention “personally and professionally rewarding” [45]. Morley et al. (2020) found increased confidence, decreased fear, and increased motivation to learn among midwives using their toolkit [47].

Adoption was assessed in two studies [45,47]. Morley et al. (2020) described the integration of their toolkit into institutional resources, e.g., a patient-facing booklet and clinician-facing training module [47]. Berndl and Khatib (2021) highlighted that their initiative was ultimately rolled out to all sonographers in their clinic [45]. Only one study assessed fidelity; DeWolff et al. (2021) reported that overall adherence to key intervention components was high [46].

### 3.2. Intellectual Impairments

In total, four interventions were adapted to address the needs of populations with intellectual impairments (i.e., difficulty learning, applying knowledge, making decisions) (Table 4). They were delivered by midwives [48,50,51,52] and doulas [49] and addressed improvements in competent and motivated human resources, effective communication, and actionable information systems. Beake et al. (2013) piloted a competency assessment tool to support midwives in providing individualized care and effective communication to pregnant patients with intellectual disabilities (ID) [48]. McGarry and team (2016) reported on a doula support intervention intended to provide advocacy, tangible assistance, emotional support, and information provision adapted to the needs of women with ID [49]. Cox and team (2024; 2021) reported on the development and testing of a toolkit and maternity passport designed to improve the experience of care for patients with ID [50,51]. The Together Toolkit provides guidance to support midwives’ delivery of high-quality respectful maternity care for women with ID, while the passport worked to improve actionable information systems by providing a paper record to patients with ID to support information sharing [50,51]. Cox and Tobutt (2024) also evaluated an awareness training for midwives co-produced with people with learning disabilities (experts-by-experience), which included pre-training materials (ground rules, written account of patient stories, educational films, Together Toolkit, Maternity Passport) and in-person content facilitated by experts-by-experience [52].

Acceptability outcomes were reported for three interventions [49,50,51,52]. McGarry et al. (2016) qualitatively assessed acceptability among patients with intellectual disabilities who received doula care; patients thought doulas were helpful, reliable, and trustworthy, and facilitated informed decisions [49]. Cox and team (2024; 2021) found that providers thought their toolkit would be useful to their practice, served their informational needs, and helped them to empower patients, and improve interprofessional relationships [50,51]. They also evaluated satisfaction among midwifery students with their learning disability awareness training, who described it positively and found the most notable aspect to be learning from people with disabilities directly. Interviews with those people (experts-by-experience) emphasized the value of reasonable adjustments in their care delivery [52].

Adoption was reported for three interventions [48,50,51,52]. Beake et al. (2013) discussed the development and piloting of their competency tool among midwives following focus group discussions to identify key themes and needs for provision of high-quality care to women with ID [48]. Cox et al. (2021) reported on their multi-phase process of developing the Together Toolkit and maternity passport, detailing how they were piloted among health care professionals to collect feedback and inform their refinement [51]. In a follow-up study, Cox and Ip (2024) assessed feasibility and made recommendations on reasonable adjustments needed to improve the implementation of resources [50]. For their midwife awareness training, Cox and Tobutt (2024) discussed the process of co-developing and co-delivering training content with “experts-by-experience” or people living with learning disabilities [52].

### 3.3. Mental Impairments

Populations with mental (psychological or behavioral) impairments were the focus of five interventions [53,54,55,56,57] (Table 4). The interventions targeted populations with a range of psychological conditions, including eating disorders [53], major depressive disorder (MDD) and/or dysthymia [54], post-traumatic stress disorder (PTSD) [57], serious mental illness (SMI) [56], and other mental health issues [55]. They employed a range of multi-disciplinary providers, including psychologists/psychiatrists [54,55,56,57], nurses [54,55,57], midwives [56,57], physicians [56,57], OBGYNs [56], social workers [54,55,57], doulas [57], depression care specialists [54], nutritionists [54], technicians [57], lactation consultants [57], pelvic floor therapists [57], and other health care providers [53].

Three of the five interventions targeting populations with mental impairments aimed to improve functional referral systems [54,55,56]. Grote et al.’s (2016) MOMCare intervention used a collaborative care model in which primary care/OB providers, a psychiatric consultant, and depression care specialist provide coordinated case management and service delivery to pregnant patients with a probable diagnosis of major depressive disorder [54]. In their study, participants were randomized to an enhanced usual care (screening, education, referral, case management and maternity support services) or MOMCare, which included additional specialized depression care coordination, brief interpersonal psychotherapy, and/or pharmacotherapy [54]. Myors and team (2014) reported on a specialist perinatal and infant mental health program where multi-disciplinary providers delivered care coordination and therapeutic services to women with mental health conditions [55]. Hauck et al. (2013) assessed patients’ experiences at a specialized childbirth and antenatal mental health clinic which utilized a multidisciplinary model to facilitate communication between obstetric, psychiatric, and primary care providers [56]. Improving competent and motivated human resources was the primary focus of two interventions [53,57]. Khan et al. (2023) reported on a sensitivity training which aimed to improve clinicians’ confidence providing respectful services to pregnant patients with eating disorders [53]. Bachner-Melman and team’s (2023) training intervention aimed to support clinicians to provide trauma-informed care for pregnant patients with a history of PTSD [57].

Among eligible maternity care intervention for populations with mental impairments, four reported on acceptability [53,54,55,56], three of which focused on the patient perspective [54,55,56]. Grote et al. (2016) found that patients in the intervention group were significantly more satisfied with the depression care they received than patients receiving usual care (*p* = 0.004) [54]. Hauck and team (2013) found a 98% satisfaction rate with their specialty clinic overall, with most patients endorsing the importance of the continuity of care it offered [56]. Myors et al. (2014) assessed satisfaction with their specialized perinatal mental health program qualitatively among patients, who expressed their reliance on the services offered and satisfaction with the relationships developed with clinicians [55]. One study assessed provider satisfaction; Khan et al. (2023) also used qualitative methods to assess satisfaction among health professionals after receiving eating disorder sensitivity training, who found the strategies offered helpful overall [53]. Only one study reported on adoption [56], one on fidelity [54], and one on penetration [57]. Hauck et al. (2013) detailed how local research recommendations and reallocation of resources led to the creation of their specialty clinic [56]. Grote (2016) reported high intervention adherence among MOMCare recipients who were more likely to engage in all intended psychotherapy sessions compared to those receiving usual care [54]. Bachner-Melman (2023) reported briefly on penetration of their intervention, noting it has expanded beyond labor wards to include other medical settings [57].

## 4. Discussion

Our scoping review is the first to systematically document lessons from the implementation of disability-inclusive maternity care. Building on a prior review that examined clinical outcomes and barriers to care [34], we aimed to understand advancements in implementing innovative approaches to improve the quality of maternity care for PwD—yet found significant evidence gaps. Despite global advocacy for disability inclusion following adoption of the UN CRPD [9,58,59], we identified only 13 articles reporting on implementation experience of 12 interventions seeking to improve maternity care for PwD. Most identified interventions addressed the provision of maternity care and health workforce capacity, and very few aimed to improve PwD’s experience of care (i.e., effective communication, respect and dignity, and emotional support). Assessment of the identified interventions focused primarily on acceptability and adoption, generally reporting high acceptability among providers and patients and emphasizing the importance of participatory development and phased introduction. Little insights documented the penetration, and none addressed the sustainability of such approaches.

Our review exposed several noteworthy gaps in implementation research on disability-inclusive maternity approaches. People with disabilities are not a monolith. Significant gaps were identified across different impairment categories. For example, none of the interventions targeted the improvement of maternity care for patients with sensory impairments (hearing or vision). This was striking, particularly given the well-documented unique barriers they face [18,21,25,31,60]. While there are policies and guidance for reasonable accommodations in some health care settings (i.e., sign language interpretation, braille) [61,62,63] there is a lack of understanding around when and how these types of accommodations are integrated, adapted, and applied in maternity care [64]. Other categories of impairment were also underrepresented, for instance, we found limited evidence on disability-inclusive maternity approaches for women with mobility impairments, or neurodivergent populations. Additional research is certainly needed to investigate how best to modify or adjust services to enable the full participation of PwD in their maternity care, regardless of their impairment.

We found that the evidence base has not been responsive to the barriers thoroughly documented in the literature for disabled populations in maternity care. Research suggests that respect and autonomy in decision making play a critical role in PwD having a positive maternity care experience, as they may face ableism and assumptions from providers who have biases and/or limited knowledge [10,14,16,27,30]. Only one study [52] integrated people with lived experience into the development and delivery of their intervention, making the disability justice movement’s decades old motto: “nothing about us without us” an exception, rather than the minimum standard that it ought to be [65,66]. Furthermore, our review found few interventions that explicitly addressed patients’ experience of care—some addressed provider competency, a cross-cutting dimension of quality with implications on both the provision and experience of care. Several surveys have found that perinatal providers in the United States and Canada receive little to no training on caring for PwD, but they express interest in incorporating it into their education to improve their comfort and capacity to provide care to women with disabilities [67,68,69,70]. Similarly, only one study [45] mentioned equipment modifications or physical resources, another cross-cutting dimension of quality, despite inaccessible infrastructure (facilities and equipment) being frequently reported as a barrier faced by PwPD [13,16,23,24].

Despite limited insights, the importance of improving functional referral systems with coordinated delivery of health services was prominent in our review. This focus is supported by growing evidence demonstrating that continuity of care plays a role in patients’ satisfaction and can be emotionally protective for those with mental health conditions [71,72,73]. Interventions addressing fragmented care delivery may be particularly important for those with disabilities given they often engage with multiple health care providers and specialists [11,12,13,14,15]. For instance, a scoping review assessing women with physical disabilities’ experience of maternity care advocated for collaborative care approaches and found that without inter-disciplinary communication and coordination, “women became the messengers of complex medical information between specialists” [13]. Several articles in our review sought to address this through actionable information systems [45,50,51].

Further assessment of innovative approaches to improve care coordination, communication, accessibility, and maternity workforce capacity is critical. Notably, only one intervention in our review introduced doula care to improve patients with intellectual disabilities’ experience of care [49]. However, research has demonstrated the impact of doula care in reducing disparities and improving respectful care, emotional support, and effective patient-provider communication among non-disabled, low-income, and racially ethnically diverse women [74,75,76]. More research is necessary to understand how doulas or other community health workers could play a similar advocacy and service delivery role for PwD, who also face challenges in communication and autonomy [14,15,21,24,30]. Similarly, though five studies focused on midwifery practitioners or trainees, most settings utilized a medical, as opposed to midwifery, model of care. The midwifery model of care is intentionally holistic and relational, prioritizing individualized, person-centered decision making [77]. As such, it could be a more suitable modality for PwD. Further investigation of the role of midwifery models of care in improving the quality of maternity care for PwD is warranted. Additionally, there is growing evidence demonstrating the potential for telehealth services and digital health tools to remove barriers to engaging with maternity care and improve a variety of health outcomes [78,79]. Their application or adaptation for pregnant patients with disabilities also warrants further exploration.

The methodological quality of the evidence base is also lacking. Across populations and domains, interventions were tested via small pilots, yielding limited insights. Many reported scant implementation outcomes and provided an incomplete picture of the intervention, exclusively focusing on either patient or provider perspectives, rarely with a multi-stakeholder health systems lens. More rigorous implementation science approaches are needed to understand how to most effectively implement interventions that address the maternal health needs of PwD. Research must move beyond documentation of intervention acceptability to share more insightful learning on how to introduce, maintain, scale-up, and ensure sustainability of disability inclusive maternity approaches.

Stakeholders around the globe have called for increased action to make health systems responsive to the needs of people with disabilities and address disparities in their health outcomes [80,81]. Our results reinvigorate these and many other calls for expanded research and programming to support patients with disabilities during pregnancy and childbirth [8,82,83,84]. While there has been increased attention to maternal health for PwD since Malouf et al.’s 2014 review [34], evidence on the implementation of interventions to improve the quality of maternity services remains limited. Our findings, paired with growing evidence on disparate maternal health outcomes and experiences among PwD, emphasize the urgent need to develop and evaluate the implementation of disability-inclusive maternal health interventions that are responsive to the needs of all PwD.

Our review has several limitations. Although we employed a systematic approach, we elected to limit our search to the peer-reviewed literature. Gray literature may provide additional insights on implementation experiences, albeit in a less scientifically rigorous format. In addition, significant heterogeneity in the way disability is defined and measured across the literature [85] may have influenced our results. To mitigate this issue, our search strategy included key terms related to the disability categories, the spectrum of impairments, and specific disabling conditions to ensure a comprehensive inquiry. In adherence to the CRPD definition [9], we included interventions targeting women with mental impairments. As with each of our defined impairment classifications, we excluded interventions for populations who experienced a new onset of impairments during the antepartum, intrapartum, or postpartum period. However, employing this criterion in practice for mental health impairments was challenging—and led to the exclusion of many articles focusing on perinatal mental health conditions that did not explicitly mention the inclusion of women with pre-existing conditions. While we found ample interventions aiming to treat the underlying mental health conditions, few aimed to focus on innovations that adapt maternal health services and support patients with pre-existing mental health impairments.

## 5. Conclusions

We synthesized the available evidence on interventions to improve the quality of maternity care for PwD. Only 13 relatively small studies targeting populations with physical, intellectual, and mental impairments were identified, and no literature on populations with sensory impairments. Interventions focused primarily on care coordination and provider competency, and few addressed patients’ experience of care. Additional research on disability-inclusive maternity care interventions, ideally involving PwD in their design and implementation, are needed to address the multifaceted institutional and interpersonal barriers they face in receiving quality health care.

## Figures and Tables

**Table 1:** Literature Eligibility Criteria.

Concept	Include	Exclude
Population	Pregnant or postpartum patients with an existing physical, sensory, mental, or intellectual impairment at onset of pregnancy	Patients who are not pregnant or beyond 12 months postpartum Patients with a pregnancy-related disability, new onset perinatal mental health disorder, or substance use disorder
Intervention	Maternity care interventions for patients with disabilities in the antenatal period, during childbirth, and/or postpartum addressing one or more domain of quality in maternal health care [41]	Interventions focused on services outside of maternity or pregnancy care Interventions focused on treatment of underlying health conditions (where the focus in not maternity care interventions/adaptations) or prevention of new disabilities during pregnancy/postpartum Interventions focused on substance use disorder, where disability or co-occurring disabling health condition is not mentioned Pharmacological interventions
Setting	Any (health care, home, and community settings)	n/a
Comparison	Any or none	n/a
Outcomes	Acceptability Adoption Fidelity Penetration Sustainability	No implementation outcomes reported.
Study Type	All studies	n/a
Article Type	Research Commentary	Protocols Case reports Dissertations or theses Abstracts/Posters Reviews
Date	Published January 2013–July 2025	Published before 2013
Languages	English, French, Spanish	All other languages

**Table 2:** Categories of Impairment.

Impairment	Definition [43]	Disabling Health Conditions ^1^
Physical	Related to physical functions and structures of the body arising from birth or due to illness or injury.	i.e.,: Absence/loss of limbs, Arthritis, Cerebral Palsy, Epilepsy, Neuromuscular disorders, SCI/Paralysis
Sensory	Related to one or more of the five senses: sight, hearing, taste, touch, or smell.	i.e.,: Blindness, Deafness, Deaf-blindness, Speech loss
Intellectual	Relates to intellectual functioning (e.g., learning, reasoning, problem solving) and adaptive behavior (conceptual, social, practical skills).	i.e.,: ADHD, ASD, Down Syndrome, FAS, Learning and Developmental disorders
Mental ^2^	Related to those actions and behaviors that an individual does to make friends and relationships, behave within accepted limits, and cope with feelings and emotions.	i.e.,: Mood, Eating, Personality, Psychotic and Trauma-related disorders

Notes: ^1^ Non-exhaustive examples of specific disabling health conditions related to impairment. ^2^ “Mental illness/mental disorder/psychiatric impairment are considered to fall under the category of mental impairment. This category also includes what is referred to as ‘psychosocial impairment’” [43]. Abbreviations: ADHD: Attention Deficit Hyperactivity Disorder, ASD: Autism Spectrum Disorder, FAS: Fetal Alcohol Syndrome, SCI: Spinal Cord Injury.

**Table 3:** Implementation Outcome Definitions.

Domains	Definition [44]	Illustrative Constructs
Acceptability	The perception among patients and/or health system actors that the intervention is agreeable or satisfactory.	Degree of satisfaction Perceived strength of the evidence bases to support intervention Perceived barriers or burden to participation Perceived ethical consequences to intervention Willingness/intention to participate in intervention Perceived benefits of the intervention
Adoption	The uptake and utilization of the intervention from the perspective of the implementer.	Decision and/or development of the intervention Readiness for implementation: Training and/or access to knowledge Readiness for implementation: Available resources, including infrastructure, supplies, materials
Fidelity	The degree to which the interventions were implemented as prescribed as intended.	Adherence to intervention protocol/process Quality of program delivery Implementing actor responsiveness or participation in the intervention
Penetration	The organization or provider perspective of the level of integration or scale of intervention within the given setting.	Scale of intervention’s saturation within the given setting or organization Intervention linkages to other programs, organizations, or systems
Sustainability	The extent to which the intervention is maintained or institutionalized within a health system or non-clinical setting from the administrator/organization perspective.	Institutional ownership of intervention Institutionalized capacity/knowledge to maintain intervention Sustained funding streams for intervention Community involvement in intervention

**Table 4:** Characteristics of Studies Identified in the Review.

Author and Date	Location	Population	QoC Domain	Description	Setting	Design	Sample
*Physical Impairment(s)*							
Berndl 2021 [45]	Canada	Pregnant w/physical disabilities	Actionable information systems	Quality improvement approach employing enhanced information systems to identify and implement adaptations to sonography services	Specialty clinic within tertiary care hospital	Cross- sectional	136 ultrasound scans from 23 patients
deWolff 2021 [46]	Denmark	Pregnant and postpartum w/chronic medical conditions ^a^	Functional referral systems	Midwife coordinated and individualized care with consultations and specialized known midwives	Tertiary care hospital	RCT	357 patients (131 intervention, 131 usual care)
Morley 2020 [47]	United Kingdom	Pregnant w/epilepsy	Evidence-based practices	Peer-reviewed maternity epilepsy shared-care toolkit for antenatal booking appointment	Multiple settings (NHS Trust ^b^)	Qualitative	4 community midwives (interviews)
*Intellectual Impairment(s)*							
Beake 2013 [48]	United Kingdom	Pregnant w/ID	Competent & motivated human resources	Competency assessment tool to support midwifery care	Tertiary care hospital within NHS Trust ^b^	Mixed methods	23 midwives (focus groups), 46 midwives (pilot and survey)
McGarry 2016 [49]	United Kingdom	Pregnant and postpartum w/ID	Effective communication	Provision of doula care: emotional support, advice and information provision, tangible assistance, and advocacy	Not specified	Qualitative	4 patients and 3 doulas (interviews)
Cox and Tobutt 2024 [52]	United Kingdom	Pregnant and postpartum w/learning disabilities	Competent & motivated human resources	Learning disability awareness training for undergraduate midwifery students (Pre-training materials and in-person content delivered by experts-by-experience ^c^)	Academic setting (midwifery school)	Mixed methods	83 midwifery students (pre-post survey), 7 experts-by-experience ^c^ (interviews)
Cox and Ip 2024; Cox 2021 [50,51]	United Kingdom	Pregnant w/learning disabilities	Actionable information systems	Development of a toolkit for maternity professionals with guidance on delivering high quality care and an accessible maternity care plan for patients	Multiple settings (NHS Trust ^b^)	2024: Mixed methods 2021: Qualitative	2024: 17 midwives and 6 parents with learning disabilities (interviews) ^d^ 2021: 16 stakeholders ^e^ (interviews), 20 providers (pilot and survey)
*Mental Impairment(s)*							
Khan 2023 [53]	United States	Pregnant w/ED	Competent & motivated human resources	Clinician sensitivity training including education and strategies for patient-centered care with de-stigmatizing language	Academic setting (medical school)	Mixed methods	115 adult health care professionals (54 intervention, 61 reference document)
Grote 2016 [54]	United States	Pregnant w/probable diagnosis of MDD and/or dysthymia	Functional referral systems	Coordination and provision of depression care	Multiple settings (Public health centers, telehealth, community)	RCT	168 eligible pregnant women ^f^ (83 intervention, 85 usual care)
Myors 2014 [55]	Australia	Pregnant and postpartum w/multiple MH issues	Functional referral systems	Coordination and provision of specialist perinatal and infant mental health care	Multiple settings (Home, health centers, public areas, telehealth)	Qualitative	11 women who were discharged from services (interviews)
Hauck 2013 [56]	Australia	Pregnant w/SMI	Functional referral systems	Childbirth and antenatal mental health clinic w/multi-disciplinary model	Specialty clinic within tertiary care hospital	Qualitative	41 women with SMI ^g^ (interviews)
Bachner-Melman 2023 [57]	Israel	Pregnant w/symptoms of PTSD	Competent & motivated human resources	Medical staff training on birth-oriented thinking, positive psychology, and trauma-informed care	Specialty clinics within tertiary care hospitals	Commentary	n/a

Abbreviations: Eating Disorders (EDs), Intellectual Disabilities (ID), Major Depressive Disorder (MDD), Mental Health (MH), Post Traumatic Stress Disorder (PTSD), Randomized Control Trial (RCT), Severe Mental Illness (SMI), National Health Service (NHS). Notes: ^a^ Chronic Medical Conditions (CMCs) were defined as “any prolonged medical condition diagnosed 6 > months before pregnancy, with continued reoccurrence and a need for medical treatment” and included multiple disabling conditions (i.e., epilepsy, multiple sclerosis, rheumatoid arthritis, cystic fibrosis, congenital malformations, injuries) [46]. ^b^ NHS Trusts are organizational units of the health system in the United Kingdom which provide maternity care and in a variety of facility and community settings. ^c^ Experts-by-experience were people with learning disabilities who contributed to the study design, development, and/or implementation [52]. ^d^ Eligible individuals were “over the age of 18 years; a pregnant woman registered or identified as having learning disabilities/a pregnant woman whose partner was registered or identified as having learning disabilities/a partner of a pregnant woman who was registered or identified as having learning disabilities; under the care of maternity services within participating NHS trust; and judged by the project midwife to have the capacity to consent to the study” [50]. ^e^ Stakeholders included health and social care professionals, parents with learning disabilities, and their informal supporters/carers [51]. ^f^ Eligible individuals were over 18 years of age, with a diagnosis of probable major depressive disorder (MDD) or diagnosis of probable dysthymia, 12–32 weeks’ gestation, who spoke English and had telephone access [54]. ^g^ SMI included diagnoses of schizophrenia, bipolar disorder, nonpsychotic major depression, borderline personality disorder, anxiety disorder, post-traumatic stress disorder, and bulimia [56].

## Data Availability

Not applicable.

## Supplementary Materials

The following supporting information can be downloaded at https://www.mdpi.com/article/10.3390/healthcare13182315/s1; Supplementary Materials S1: Search Strategy. Supplementary Materials S2: PRISMAChecklist.

## Abbreviations

The following abbreviations are used in this manuscript:

CRPD	Convention on the Rights of Persons with Disabilities
ED	Eating Disorders
ICF	International Classification on Functioning and Disability
ID	Intellectual Disabilities
MDD	Major Depressive Disorder
MH	Mental Health
NHS	National Health Service
OB	Obstetrician
OBGYN	Obstetrics and Gynecology
PTSD	Post-Traumatic Stress Disorder
RCT	Randomized Controlled Trial
SMI	Severe Mental Illness
US	United States
UN	United Nations
WHO	World Health Organization
PwD	People with Disabilities
PwPD	People with Physical Disabilities

## References

[1] World Health Organization, World Bank (2011). World Report on Disability.

[2] Signore C., Davis M., Tingen C.M., Cernich A.N. (2021). The Intersection of Disability and Pregnancy: Risks for Maternal Morbidity and Mortality. J. Women’s Health.

[3] Horner-Johnson W., Darney B.G., Kulkarni-Rajasekhara S., Quigley B., Caughey A.B. (2016). Pregnancy among US Women: Differences by Presence, Type, and Complexity of Disability. Am. J. Obstet. Gynecol..

[4] Akobirshoev I., Vetter M., Horner-Johnson W., Lomerson N., Moore Simas T.A., Mitra M. (2024). Severe Maternal Morbidity by Disability Status and Type in the United States. O&G Open.

[5] Lo H.W.J., Poston L., Wilson C.A., Sheehan R., Sethna V. (2025). Pregnancy and Postnatal Outcomes for Women with Intellectual Disability and Their Infants: A Systematic Review. Midwifery.

[6] Gleason J.L., Grewal J., Chen Z., Cernich A.N., Grantz K.L. (2021). Risk of Adverse Maternal Outcomes in Pregnant Women with Disabilities. JAMA Netw. Open.

[7] Shea L., Sadowsky M., Tao S., Rast J., Schendel D., Chesnokova A., Headen I. (2024). Perinatal and Postpartum Health Among People With Intellectual and Developmental Disabilities. JAMA Netw. Open.

[8] Kuper H., Rodriguez D. (2025). Disparities in Maternity Care for Disabled Women in the UK.

[9] United Nations General Assembly (2007). Convention on the Rights of Persons with Disabilities.

[10] Hall J., Hundley V., Collins B., Ireland J. (2018). Dignity and Respect during Pregnancy and Childbirth: A Survey of the Experience of Disabled Women. BMC Pregnancy Childbirth.

[11] Tarasoff L.A., Lunsky Y., Welsh K., Proulx L., Havercamp S.M., Parish S.L., Brown H.K. (2023). Unmet Needs, Limited Access: A Qualitative Study of Postpartum Health Care Experiences of People with Disabilities. J. Adv. Nurs..

[12] Hill-Thomas A., Brown R., Kinser P.A. (2025). The Care Experiences of Pregnant Women with Serious Mental Illness: A Scoping Review. J. Am. Psychiatr. Nurses Assoc..

[13] Blair A., Cao J., Wilson A., Homer C. (2022). Access to, and Experiences of, Maternity Care for Women with Physical Disabilities: A Scoping Review. Midwifery.

[14] Seiedzadeh M., Khanjani M.S., Abdi K., Latifian M. (2025). Content Analysis of Barriers to Delivering Maternity Care to Women with Physical Disabilities: A Qualitative Study. BMC Public Health.

[15] Saeed G., Brown H.K., Lunsky Y., Welsh K., Proulx L., Havercamp S., Tarasoff L.A. (2022). Barriers to and Facilitators of Effective Communication in Perinatal Care: A Qualitative Study of the Experiences of Birthing People with Sensory, Intellectual, and/or Developmental Disabilities. BMC Pregnancy Childbirth.

[16] Ven C., Marella M., Vaughan C., Slade S., Devine A. (2025). Factors Influencing the Capacity of Healthcare Providers to Deliver Disability-Inclusive Maternity Care Services: A Scoping Review. Midwifery.

[17] Moore L., Foley S., Larkin F. (2025). Understanding the Experiences of Receiving and Providing Maternity Care for Autistic Adults: A Multi-Perspectival Interpretative Phenomenological Analysis Study. Autism.

[18] Panko T.L., Cuculick J., Albert S., Smith L.D., Cooley M.M., Herschel M., Mitra M., McKee M. (2023). Experiences of Pregnancy and Perinatal Healthcare Access of Women Who Are Deaf: A Qualitative Study. BJOG Int. J. Obstet. Gynaecol..

[19] Elliott J.K., Buchanan K., Bayes S. (2024). The Neurodivergent Perinatal Experience—A Systematic Literature Review on Autism and Attention Deficit Hyperactivity Disorder. Women Birth.

[20] Collins B., Hall J., Hundley V., Ireland J. (2023). Effective Communication: Core to Promoting Respectful Maternity Care for Disabled Women. Midwifery.

[21] Ratakonda S., Panko T.L., Albert S., Smith L.D., Cooley M.M., Mitra M., McKee M. (2025). Wait, What? What’s Going On?—Pregnancy Experiences of Deaf and Hard of Hearing Mothers Who Do Not Sign. Birth.

[22] Nakatabira M., Ekirapa-Kiracho E., Aanyu C., Tan H.-L., Apolot R.R., Zia N., Kajungu D., Bachani A.M., Morgan R. (2025). Improving Access to Skilled Maternal Health Services among Pregnant Women with Disabilities in Uganda: What Are Disability-Responsive Maternal Health Services?. SSM—Health Syst..

[23] Iezzoni L.I., Wint A.J., Smeltzer S.C., Ecker J.L. (2015). Physical Accessibility of Routine Prenatal Care for Women with Mobility Disability. J. Women’s Health.

[24] Kalpakjian C.Z., Mulenga L., McIntosh S.M., Kreschmer J.M., Parten R., Haapala H., Langen E.S., Rosenblum S.A.S., Pazhyanur S., Carlson S. (2025). Pregnancy and Physical Disability: A Scoping Review. Women’s Health.

[25] Diaminti A., Sarantaki A., Gourounti K., Lykeridou A. (2021). Perinatal Care in Women with Vision Disorders: A Systematic Review. Maedica.

[26] Evans M., Tarasoff L.A., Lunsky Y., Welsh K., Proulx L., Havercamp S.M., Parish S.L., Brown H.K. (2024). Disability Justice and Collective Access to Labour and Delivery Care: A Qualitative Study. BMC Pregnancy Childbirth.

[27] Smeltzer S.C., Mitra M., Long-Bellil L., Iezzoni L.I., Smith L.D. (2018). Obstetric Clinicians’ Experiences and Educational Preparation for Caring for Pregnant Women with Physical Disabilities: A Qualitative Study. Disabil. Health J..

[28] Reichard A., Alvarado M., Ruiz S., King T., Cruz T., Davis M., Wallace J. (2021). Disability and Pregnancy: Research from NIDILRR and NICHD.

[29] Mitra M., Akobirshoev I., Moring N.S., Long-Bellil L., Smeltzer S.C., Smith L.D., Iezzoni L.I. (2017). Access to and Satisfaction with Prenatal Care Among Pregnant Women with Physical Disabilities: Findings from a National Survey. J. Women’s Health.

[30] Mitra M., Smith L.D., Smeltzer S.C., Long-Bellil L.M., Sammet Moring N., Iezzoni L.I. (2017). Barriers to Providing Maternity Care to Women with Physical Disabilities: Perspectives from Health Care Practitioners. Disabil. Health J..

[31] Makeroufa C., Diamanti A. (2024). Exploring Perinatal Care and Birth Experiences in Women with Visual Impairment: A Retrospective Study. Med. Int..

[32] American College of Obstetricians and Gynecologists (2025). Access to Obstetric and Gynecologic Care for Patients with Disabilities.

[33] World Health Organization (2024). Health Equity for Persons with Disabilities: A Guide for Action.

[34] Malouf R., Redshaw M., Kurinczuk J.J., Gray R. (2014). Systematic Review of Heath Care Interventions to Improve Outcomes for Women with Disability and Their Family during Pregnancy, Birth and Postnatal Period. BMC Pregnancy Childbirth.

[35] World Health Organization (2001). International Classification of Functioning Disability and Health (ICF).

[36] Schaefer N. (2015). Disability Search Tips and Resources. Med. Ref. Serv. Q..

[37] Ioerger M., Flanders R.M., Goss K.D., Turk M.A. (2019). Developing a Systematic Search Strategy Related to People with Disability: A Brief Report Testing the Utility of Proposed Disability Search Terms in a Search about Opioid Use. Disabil. Health J..

[38] Walsh E.S., Peterson J.J., Judkins D.Z. (2014). Searching for Disability in Electronic Databases of Published Literature. Disabil. Health J..

[39] Clifford S., Mary M., Interventions to Improve the Quality of Maternity Care for People with Disabilities: A Scoping Review Open Science Framework (OSF) Registries. https://osf.io/j7qdb.

[40] Tricco A.C., Lillie E., Zarin W., O’Brien K.K., Colquhoun H., Levac D., Moher D., Peters M.D.J., Horsley T., Weeks L. (2018). PRISMA Extension for Scoping Reviews (PRISMA-ScR): Checklist and Explanation. Ann. Intern. Med..

[41] World Health Organization (2016). Standards for Improving Quality of Maternal and Newborn Care in Health Facilities.

[42] Veritas Health Innovation (2024). Covidence Systematic Review Software. www.covidence.org.

[43] UNRWA (2013). Promoting the Rights of Persons with Disabilities: Disability Toolkit: Classifying Disability.

[44] Proctor E., Silmere H., Raghavan R., Hovmand P., Aarons G., Bunger A., Griffey R., Hensley M. (2011). Outcomes for Implementation Research: Conceptual Distinctions, Measurement Challenges, and Research Agenda. Adm. Policy Ment. Health.

[45] Berndl A., Khatib S. (2021). Fetal Ultrasound Challenges and Solutions for Scanning Pregnant People with Physical Disabilities: A Two-Year Initiative for Adaptation. J. Obstet. Gynaecol. Can..

[46] de Wolff M.G., Midtgaard J., Johansen M., Rom A.L., Rosthøj S., Tabor A., Hegaard H.K. (2021). Effects of a Midwife-Coordinated Maternity Care Intervention (ChroPreg) vs. Standard Care in Pregnant Women with Chronic Medical Conditions: Results from a Randomized Controlled Trial. Int. J. Environ. Res. Public Health.

[47] Morley K. (2020). Reducing Risks for Pregnant Women with Epilepsy: A Qualitative Study Exploring Experiences of Using a Toolkit at the Antenatal Booking Appointment. Epilepsy Behav..

[48] Beake S., Clark L., Turner T., Bick D. (2013). A Mixed Methods Study to Develop and Pilot a Competency Assessment Tool to Support Midwifery Care of Women with Intellectual Disabilities. Nurse Educ. Today.

[49] McGarry A., Stenfert Kroese B., Cox R. (2016). How Do Women with an Intellectual Disability Experience the Support of a Doula During Their Pregnancy, Childbirth and After the Birth of Their Child?. J. Appl. Res. Intellect. Disabil..

[50] Cox A., Ip A., Watkin S., Matuska G., Bunford S., Gallagher A., Taylor C. (2024). Implementing and Evaluating Resources to Support Good Maternity Care for Parents with Learning Disabilities: A Qualitative Feasibility Study in England. Midwifery.

[51] Cox A., Parsons T., Watkin S., Gallagher A. (2021). Supporting the Delivery of Good Maternity Care for Parents with Learning Disabilities. Midwifery.

[52] Cox A., Tobutt D., Harris J., Watkin S., Eynon C., Matuska G. (2024). Learning Disability Awareness Training for Undergraduate Midwifery Students: Multi-Method Evaluation of a Co-Produced and Co-Delivered Educational Intervention in England. Nurse Educ. Today.

[53] Khan Z.A., Lilly C.L., DeFazio C., Claydon E.A. (2023). “It Is More Isolating to Patients If You Aren’t Familiar with the Resources”: A Pilot Test of a Clinician Sensitivity Training on Eating Disorders in Pregnancy. BMC Med. Educ..

[54] Grote N.K., Katon W.J., Russo J.E., Lohr M.J., Curran M., Galvin E., Carson K. (2016). A Randomized Trial of Collaborative Care for Perinatal Depression in Socioeconomically Disadvantaged Women: The Impact of Comorbid Posttraumatic Stress Disorder. J. Clin. Psychiatry.

[55] Myors K.A., Schmied V., Johnson M., Cleary M. (2014). “My Special Time”: Australian Women’s Experiences of Accessing a Specialist Perinatal and Infant Mental Health Service. Health Soc. Care Community.

[56] Hauck Y., Allen S., Ronchi F., Faulkner D., Frayne J., Nguyen T. (2013). Pregnancy Experiences of Western Australian Women Attending a Specialist Childbirth and Mental Illness Antenatal Clinic. Health Care Women Int..

[57] Bachner-Melman R., Haim-Dahan R., Zohar A.H. (2023). “Women Friendly”: A Childbirth Preparation Intervention in Israel for Women with Symptoms of Post-Traumatic Stress Disorder. Int. J. Environ. Res. Public Health.

[58] Global Disability Summit (2025). Global Disability Inclusion Report: Accelerating Disability Inclusion in a Changing and Diverse World.

[59] Stein M.A., Stein P.J., Weiss D., Lang R. (2009). Health Care and the UN Disability Rights Convention. Lancet.

[60] Adigun O.T., Akinrinoye O., Obilor H.N. (2021). Including the Excluded in Antenatal Care: A Systematic Review of Concerns for D/Deaf Pregnant Women. Behav. Sci..

[61] U.S. Department of Justice Civil Rights Division ADA Requirements: Effective Communication. https://www.ada.gov/resources/effective-communication/.

[62] U.S. Department of Justice Civil Rights Division ADA Business Brief: Communicating with People Who Are Deaf or Hard of Hearing in Hospital Settings. https://www.ada.gov/resources/business-brief-hospital/.

[63] U.S. Department of Justice Civil Rights Division Access to Medical Care for Individuals with Mobility Disabilities. https://www.ada.gov/resources/medical-care-mobility/.

[64] MacLellan J., McNiven A., Kenyon S. (2024). Provision of Interpreting Support for Cross-Cultural Communication in UK Maternity Services: A Freedom of Information Request. Int. J. Nurs. Stud. Adv..

[65] Charlton J. (1998). Nothing About Us Without Us: Disability Oppression and Empowerment.

[66] Rios D., Magasi S., Novak C., Harniss M. (2016). Conducting Accessible Research: Including People with Disabilities in Public Health, Epidemiological, and Outcomes Studies. Am. J. Public Health.

[67] Bekdache G.N., Berndl A. (2019). Women with Physical Disability in Pregnancy Resident Education: A National Survey as a Needs Assessment for Curriculum Improvement in Obstetrics and Gynaecology in Canada. BMJ Open.

[68] Taouk L.H., Fialkow M.F., Schulkin J.A. (2018). Provision of Reproductive Healthcare to Women with Disabilities: A Survey of Obstetrician-Gynecologists’ Training, Practices, and Perceived Barriers. Health Equity.

[69] Carlson S., Aitelli A., Dotters-Katz S., Kalpakjian C. (2025). Obstetrics and Gynecology Resident Comfort in Caring for Pregnant People with Physical Disabilities. Am. J. Perinatol..

[70] Leddy M.A., Jones C., Morgan M.A., Schulkin J. (2009). Eating Disorders and Obstetric-Gynecologic Care. J. Women’s Health.

[71] Cummins A., Eaves T., Newnham E., Melov S., Hilsabeck C., Baird K., Prussing E., Pasupathy D. (2025). The Continuity Relationship Makes Caring for Women with Anxiety and Depression Easier, but It Is Also a Heavy Responsibility. Women Birth.

[72] Cibralic S., Pickup W., Diaz A.M., Kohlhoff J., Karlov L., Stylianakis A., Schmied V., Barnett B., Eapen V. (2023). The Impact of Midwifery Continuity of Care on Maternal Mental Health: A Narrative Systematic Review. Midwifery.

[73] Barr K.R., Nguyen T.A., Pickup W., Cibralic S., Mendoza Diaz A., Barnett B., Eapen V. (2024). Perinatal Continuity of Care for Mothers with Depressive Symptoms: Perspectives of Mothers and Clinicians. Front. Psychiatry.

[74] Kozhimannil K.B., Vogelsang C.A., Hardeman R.R., Prasad S. (2016). Disrupting the Pathways of Social Determinants of Health: Doula Support during Pregnancy and Childbirth. J. Am. Board. Fam. Med..

[75] Mallick L.M., Thoma M.E., Shenassa E.D. (2022). The Role of Doulas in Respectful Care for Communities of Color and Medicaid Recipients. Birth.

[76] Louis-Jacques A.F., Applequist J., Perkins M., Williams C., Joglekar R., Powis R., Daniel A., Wilson R. (2024). Florida Doulas’ Perspectives on Their Role in Reducing Maternal Morbidity and Health Disparities. Women’s Health Issues.

[77] International Confederation of Midwives (2025). Philosophy and Model of Midwifery Care.

[78] Gebreyes K., Nelson H., Punch M., Bhatt J., Keita M., Chang C. (2024). Maternal Health Inequities Persist. Can Digital Tools Be Part of the Solution?.

[79] Atkinson J., Hastie R., Walker S., Lindquist A., Tong S. (2023). Telehealth in Antenatal Care: Recent Insights and Advances. BMC Med..

[80] World Health Organization (2022). Global Report on Health Equity for Persons with Disabilities.

[81] Nguyen T.V., Kane S. (2024). Towards an Agenda of Action and Research for Making Health Systems Responsive to the Needs of People with Disabilities. Lancet Reg. Health—West. Pac..

[82] Brown H.K., Mitra M. (2022). Improved Obstetric Care for People with Disabilities: An Urgent Call for Accessibility and Inclusion. J Women’s Health.

[83] Office of the Surgeon General (OSG) (2020). The Surgeon General’s Call to Action to Improve Maternal Health.

[84] Make Mothers Matter (2025). Supporting Mothers with Disabilities: A Call for Enhanced Care and Resources.

[85] Bills K.L., Mills B. (2022). Limitations When Conducting Quantitative Disability Research. J. Res. Initiat..

