# Supplementary material for: Learning from the Implementation of Disability-Inclusive Maternity Care: A Scoping Review
Source: Healthcare (Basel). 2025 Sep 16;13(18):2315. doi: 10.3390/healthcare13182315 (PMC12470062; doi:10.3390/healthcare13182315)
Supplement: Supplementary file 1 [file healthcare-13-02315-s001.zip › healthcare-3817667-supplementary/S1_Search Strategy.pdf]

Supplementary Material S1:

PubMed Search Strategy:

#1	"Pregnancy"[Mesh] OR "Pregnant women"[tiab] OR "Pregnant woman"[tiab] OR "pregnant person"[tiab] OR "pregnant people"[tiab] OR "pregnant girl*"[tiab] OR "pregnant adolescent*"[tiab] OR "pregnant teen*"[tiab] OR "Child-bearing women"[tiab:~3] OR "child-bearing woman"[tiab:~3] OR "child-bearing person"[tiab:~3] OR "child-bearing people"[tiab:~3] OR "child-bearing girl"[tiab:~3] OR "child-bearing adolescent"[tiab:~3] OR "child-bearing teen"[tiab:~3] OR "Breastfeeding women"[tiab] OR "breastfeeding woman"[tiab] OR "breastfeeding person"[tiab:~3] OR "breastfeeding people"[tiab] OR "breastfeeding girl"[tiab:~3] OR "breastfeeding adolescent*"[tiab] OR "breastfeeding teen"[tiab:~3] OR "Breast feeding women"[tiab] OR "breast feeding woman"[tiab] OR "breast feeding person"[tiab:~3] OR "breast feeding people"[tiab:~3] OR "breast feeding girl"[tiab:~3] OR "breast feeding adolescent*"[tiab] OR "breast feeding teen"[tiab:~3] OR "postpartum women"[tiab] OR "postpartum woman"[tiab] OR "postpartum person"[tiab:~3] OR "postpartum people"[tiab] OR "postpartum girl"[tiab:~3] OR "postpartum adolescent*"[tiab] OR "postpartum teen*"[tiab] OR "post partum women"[tiab] OR "post partum woman"[tiab] OR "post partum person"[tiab:~3] OR "post partum people"[tiab:~3] OR "post partum girl"[tiab:~3] OR "post partum adolescent"[tiab:~3] OR "post partum teen"[tiab:~3] OR "child bearing women"[tiab] OR "child bearing woman"[tiab] OR "child bearing person"[tiab:~3] OR "child bearing people"[tiab:~3] OR "child bearing girl"[tiab:~3] OR "child bearing adolescent"[tiab:~3] OR "child bearing teen"[tiab:~3] OR "birthing people"[tiab] OR "birthing person"[tiab] OR parent*[tiab]
#2	"Disabled persons"[Mesh] OR "amputees"[Mesh] OR "persons with hearing impairments"[Mesh] OR "visually impaired persons"[Mesh] OR "homebound persons"[Mesh] OR "disabled person*"[tiab] or "disab*"[tiab] OR "amputee*"[tiab] OR "hearing impaired"[tiab] OR "visually impaired"[tiab] OR "activities of daily living"[Mesh] OR "developmental disabilities"[Mesh] OR "developmental disabilit*"[tiab] OR "Mental retardation"[tiab] OR "mental* retard*"[tiab] OR "mobility limitation"[mesh] OR "mobility limitation*"[tiab] OR "dependent ambulation"[mesh] OR "dependent ambulation*"[tiab] OR "paraplegia"[mesh] OR "paraplegia"[tiab] OR "quadriplegia"[mesh] OR "quadriplegia"[tiab] OR "hearing loss"[Mesh] OR "hearing loss"[tiab] OR "blindness"[mesh] OR "blindness"[tiab] OR "vision disorders"[mesh] OR "vision disorder*"[tiab] OR "self-help devices"[mesh] OR "self-help device*"[tiab] OR "assistive technolog*"[tiab] OR "functional limitation*"[tiab] OR "activity limitation*"[tiab] OR "mobility impairment*"[tiab] OR "vision impairment*"[tiab] OR "hearing impairment*"[tiab] OR "cognitive impairment*"[tiab] OR "intellectual disabilit*"[tiab] OR "participation limitation*"[tiab] OR "Spinal Cord Injuries"[Mesh] or "spinal cord injur*"[tiab] OR "SCI"[tiab] OR "Amputation, surgical"[Mesh] or "amputation*"[tiab] OR "Cerebral palsy"[mesh] OR "cerebral palsy"[tiab] OR "Spina bifida"[tiab] OR "Spinal dysraphism"[mesh] OR "multiple sclerosis"[mesh] or "multiple sclerosis"[tiab] OR "amyotrophic lateral sclerosis"[mesh] OR "amyotrophic lateral sclerosis"[tiab] OR "ALS"[tiab] OR "Brain injuries, traumatic"[Mesh] OR "Traumatic brain injur*"[tiab] OR "brain concussion"[mesh] OR "concussion*"[tiab] OR "muscular dystrophies"[mesh] OR "muscular dystroph*"[tiab] OR "stroke"[mesh] OR "stroke*"[tiab] OR "Attention deficit disorder with hyperactivity"[mesh] OR "Attention deficit disorder with hyperactivity"[tiab] OR "ADHD"[tiab] OR "Muscular atrophy, spinal"[mesh] OR "spinal muscular atroph*"[tiab] OR "muscular diseases"[mesh] OR "muscular disease*"[tiab] "myopath*"[tiab] OR "autism spectrum disorder"[mesh] OR "autism spectrum disorder*"[tiab] OR "ASD"[tiab] OR "Down syndrome"[mesh] OR "down* syndrome"[tiab] OR "peripheral nervous system diseases"[mesh] OR "peripheral nervous system disease*"[tiab] OR "peripheral neuropath*"[tiab] OR "paralysis"[tiab] OR "sensory disabil*" [tiab] OR "epilepsy"[mesh] OR "epilepsy"[tiab] OR "epileptic"[tiab] OR "arthritis"[tiab]

	OR “migraine disorder*”[tiab] OR “migraine*”[tiab] OR “chronic headache*”[tiab] OR “people with disabilit*”[tiab] OR “special needs”[tiab] OR “deaf”[tiab] OR “deafness”[tiab] OR “learning disorder”[tiab:~3] OR “learning disorders”[tiab:~3] OR “learning disability”[tiab:~3] OR “learning disabilities”[tiab:~3] OR “learning handicap”[tiab:~3] OR “physical disability”[tiab:~3] OR “physical disabilities”[tiab:~3] OR “physically disabled”[tiab:~3]
#3	“Mental disorders”[Mesh] OR “persons with mental disabilities”[tiab] OR “mentally disabled person*”[tiab] OR “mentally disabled people”[tiab] OR “mentally ill person*”[tiab] OR “mentally ill people”[tiab] OR “mental disorders”[mesh] OR “mental disorder*”[tiab] OR “anxiety disorders”[mesh] OR “anxiety disorder*”[tiab] OR “Affective disorders, psychotic”[mesh] OR “Depressive disorder, major”[Mesh] OR “Schizophrenia spectrum and other psychotic disorders”[Mesh] OR “Feeding and eating disorders”[mesh] OR “schizophrenia”[tiab] or “schizophrenic”[tiab] or “psychosis”[tiab] or “psychotic”[tiab] OR “eating disorder*”[tiab] or “disordered eating”[tiab] or “anorexia nervosa”[tiab] or “bulimia”[tiab] or “binge eating”[tiab] OR “psychiatric disabilit*”[tiab] OR “mental health disabilit*”[tiab] OR “mental health impairment*”[tiab] OR "serious mental"[tiab:~3] OR "severe mental"[tiab:~3] OR "major mental"[tiab:~3] OR "chronic mental"[tiab:~3] OR "long-term mental"[tiab:~3] OR "’persistent mental"[tiab:~3] OR "serious depression"[tiab:~3] OR "severe depression"[tiab:~3] OR "serious depressive"[tiab:~3] OR "severe depressive"[tiab:~3] OR "major depression"[tiab:~3] OR "major depressive"[tiab:~3] OR "chronic depression"[tiab:~3] OR "chronic depressive"[tiab:~3] OR "long-term depressive"[tiab:~3] OR "long-term depression"[tiab:~3] OR "persistent depression"[tiab:~3] OR "persistent depressive"[tiab:~3] OR "bipolar depression"[tiab:~3] OR "bipolar depressive"[tiab:~3] OR "bipolar disorder"[tiab:~3] OR "bipolar disorders"[tiab:~3] OR "manic depression"[tiab:~3] OR "manic depressive"[tiab:~3] OR “stress disorders, post-traumatic"[mesh] OR “PTSD”[tiab] OR “mood disorder*”[tiab] OR “dissociative disorder*”[tiab] OR “obsessive compulsive disorder*”[tiab]
#4	“Maternal Health”[Mesh] OR “Maternal health”[tiab] OR maternal health services[MeSH Major Topic] OR “perinatal care”[mesh] OR “prenatal care”[Mesh] OR “obstetrics”[mesh] OR “obstetrics”[tiab] OR “abortion, induced”[mesh] OR "Health Services for Persons with Disabilities"[Mesh] OR “maternal health care”[tiab] OR “maternal health service*”[tiab] OR “maternal health consult”[tiab:~3] OR “maternal health counsel”[tiab:~3] OR “maternal health education”[tiab] OR “obstetric care”[tiab] OR “obstetric service*”[tiab] OR “obstetric consult*”[tiab] OR “obstetric counsel*”[tiab] OR “obstetric education”[tiab] OR “prenatal care”[tiab] OR “prenatal service*”[tiab] OR “prenatal consult*”[tiab] OR “prenatal counsel*”[tiab] OR “prenatal education”[tiab] OR “prenatal visit*”[tiab] OR “pre-natal care”[tiab] OR “pre-natal service*”[tiab] OR “pre-natal consult*”[tiab] OR “pre-natal counsel*”[tiab] OR “pre-natal education”[tiab] OR “pre-natal visit*”[tiab] OR “antenatal care”[tiab] OR “antenatal service*”[tiab] OR “antenatal consult*”[tiab] OR “antenatal counsel*”[tiab] OR “antenatal education”[tiab] OR “antenatal visit*”[tiab] OR “postnatal care”[tiab] OR “postnatal service*”[tiab] OR “postnatal consult*”[tiab] OR “postnatal counsel*”[tiab] OR “postnatal education”[tiab] OR “postnatal visit*”[tiab] OR “post natal care”[tiab] OR “post natal service*”[tiab] OR “post natal consult*”[tiab] OR “post natal counsel*”[tiab] OR “post natal education”[tiab] OR “post natal visit*”[tiab] OR “puerperium care”[tiab] OR “puerperium service”[tiab:~3] OR “puerperium consult”[tiab:~3] OR “puerperium counsel”[tiab:~3] OR “puerperium education”[tiab:~3] OR “puerperium visit”[tiab:~3] OR “perinatal care”[tiab] OR “perinatal service*”[tiab] OR “perinatal consult*”[tiab] OR “perinatal counsel*”[tiab] OR “perinatal education”[tiab] OR “perinatal visit*”[tiab] “pregnan* care”[tiab] OR “pregnan* service*”[tiab] OR “pregnan* consult*”[tiab] OR “pregnan* counsel*”[tiab] OR “pregnan* education”[tiab] OR “pregnancy visit*”[tiab] “maternity care”[tiab] OR “maternity service*”[tiab] OR “maternity consult*”[tiab] OR “maternity counsel*”[tiab] OR “maternity education”[tiab] OR “maternity visit*”[tiab] OR “intrapartum care”[tiab] OR “intrapartum

	service*"[tiab] OR "intrapartum consult"[tiab:~3] OR "intrapartum counsel*"[tiab] OR "intrapartum education"[tiab:~3] OR "intra partum care"[tiab:~3] OR "intra partum service"[tiab:~3] OR "intra partum consult"[tiab:~3] OR "intra partum counsel"[tiab:~3] OR "intra partum education"[tiab:~3] OR "intrapartum visit"[tiab:~3] OR "childbirth care"[tiab] OR "childbirth service*"[tiab] OR "childbirth consult"[tiab:~3] OR "childbirth counsel*"[tiab] OR "childbirth education"[tiab] OR "labor care"[tiab] OR "labor service*"[tiab] OR "labor consult"[tiab:~3] OR "labor counsel"[tiab:~3] OR "labor education"[tiab:~3] OR "labour care"[tiab] OR "labour service*"[tiab] OR "labour consult"[tiab:~3] OR "labour counsel"[tiab:~3] OR "labour education"[tiab:~3] OR "abortion care"[tiab] OR "abortion service*"[tiab] OR "abortion consult*"[tiab] OR "abortion counsel*"[tiab] OR "abortion education"[tiab] OR "abortion visit"[tiab] OR "delivery care"[tiab] OR "delivery service*"[tiab] OR "delivery consult*"[tiab] OR "delivery counsel*"[tiab] OR "delivery education"[tiab] OR "postpartum care"[tiab] OR "postpartum service*"[tiab] OR "postpartum consult*"[tiab] OR "postpartum counsel*"[tiab] OR "postpartum education"[tiab] OR "postpartum visit*"[tiab] OR "post-partum care"[tiab] OR "post-partum service"[tiab:~3] OR "post-partum consult"[tiab:~3] OR "post-partum counsel*"[tiab] OR "post-partum education"[tiab:~3] OR "post-partum visit*"[tiab] OR "doula care"[tiab] OR "doula support"[tiab] OR "miscarriage care"[tiab] OR "miscarriage service*"[tiab] OR "miscarriage consult"[tiab:~3] OR "miscarriage counsel"[tiab:~3] OR "pregnancy loss care"[tiab:~3] OR "pregnancy loss service"[tiab:~3] OR "pregnancy loss consult"[tiab:~3] OR "pregnancy loss counsel"[tiab:~3] OR maternity[tiab]
#5	#2 OR #3
#6	#1 AND #4 AND #5
#7	Filters applied: Date (2013-2024)
#8	Filters applied: Language (English, French, Spanish)
